# SMC5/6 Promotes Replication Fork Stability via Negative Regulation of the COP9 Signalosome

**DOI:** 10.3390/ijms25020952

**Published:** 2024-01-12

**Authors:** Michelle J. Xu, Philip W. Jordan

**Affiliations:** 1 Department of Biochemistry and Molecular Biology, Johns Hopkins University Bloomberg School of Public Health, Baltimore, MD 21205, USA; 2 Department of Biochemistry and Molecular Biology, Uniformed Services University of the Health Sciences, Bethesda, MD 20814, USA

**Keywords:** structural maintenance of chromosomes, SMC5/6, DNA replication, DNA damage, DNA repair, embryonic stem cells

## Abstract

It is widely accepted that DNA replication fork stalling is a common occurrence during cell proliferation, but there are robust mechanisms to alleviate this and ensure DNA replication is completed prior to chromosome segregation. The SMC5/6 complex has consistently been implicated in the maintenance of replication fork integrity. However, the essential role of the SMC5/6 complex during DNA replication in mammalian cells has not been elucidated. In this study, we investigate the molecular consequences of SMC5/6 loss at the replication fork in mouse embryonic stem cells (mESCs), employing the auxin-inducible degron (AID) system to deplete SMC5 acutely and reversibly in the defined cellular contexts of replication fork stall and restart. In SMC5-depleted cells, we identify a defect in the restart of stalled replication forks, underpinned by excess MRE11-mediated fork resection and a perturbed localization of fork protection factors to the stalled fork. Previously, we demonstrated a physical and functional interaction of SMC5/6 with the COP9 signalosome (CSN), a cullin deneddylase that enzymatically regulates cullin ring ligase (CRL) activity. Employing a combination of DNA fiber techniques, the AID system, small-molecule inhibition assays, and immunofluorescence microscopy analyses, we show that SMC5/6 promotes the localization of fork protection factors to stalled replication forks by negatively modulating the COP9 signalosome (CSN). We propose that the SMC5/6-mediated modulation of the CSN ensures that CRL activity and their roles in DNA replication fork stabilization are maintained to allow for efficient replication fork restart when a replication fork stall is alleviated.

## 1. Introduction

In proliferating cells, genome duplication is a major genotoxic-stress-inducing event. DNA replication machinery must counteract intrinsic obstacles within the genome, such as repetitive sequences, secondary structure, and DNA lesions. Impediments to DNA synthesis also occur in the form of nucleotide deficits, superhelical strain at replication termination regions, and obstruction by transcription machinery [1]. These challenges to DNA synthesis lead to temporary stalling of the replication fork, during which the decoupling of replicative helicase and polymerase generates stretches of single-stranded DNA (ssDNA) [2,3]. Replication protein A (RPA) binds and stabilizes the ssDNA at the fork [4,5]. The stalled fork is detected by the ataxia telangiectasia and Rad3-related (ATR) kinase, which triggers a signaling cascade that arrests cell cycle progression and initiates DNA repair mechanisms [6,7].

**Figure 1:**
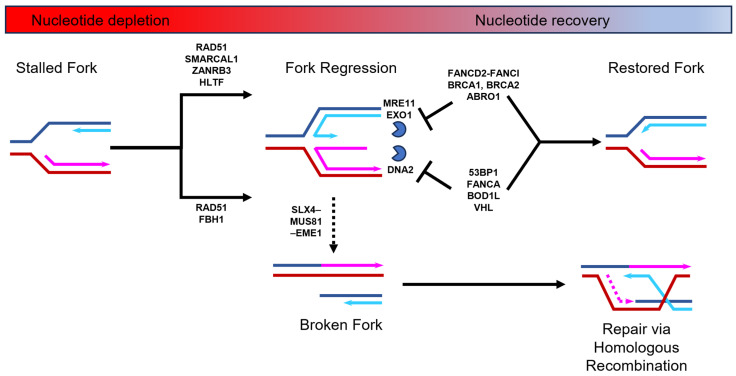
DNA replication fork stalling, regression, and restart during nucleotide depletion and recovery. When nucleotide levels drop, DNA replication forks stall and regress to form a “chicken foot” structure. This is stimulated by two alternative pathways that both require RAD51 nucleofilament formation on the exposed nascent DNA of the extruded arm. One pathway requires the action of the FBH1 helicase and the other requires the SMARCAL1, ZANRB3, and HLTF DNA translocases. This regressed fork is susceptible to nuclease degradation via MRE11, EXO1, or DNA2 nucleases (depicted as blue partial circle). To combat this, DNA replication fork protection factors that are specific to each alternative pathway are loaded (e.g., 53BP1 or FANCD2-FANCI). Prolonged nucleotide deficiency may lead to the replication fork being broken by the SLX4–MUS81–EME1 endonuclease complex and subsequent repair via homologous recombination. See relevant text for further details.

Nucleotide depletion typically causes stalled DNA replication forks to undergo a process known as regression [8,9]. DNA replication fork regression involves the unwinding of a portion of the already replicated DNA that results in the formation of a four-stranded DNA structure known as a “chicken foot”, which is structurally similar to a Holliday junction (Figure 1). This regression process can be stimulated by two alternative remodeling pathways. One pathway requires the SNF2-family ATPase-dependent DNA translocases of SMARCAL1, ZRANB3, and HLTF [10], and the other relies on the F-box DNA helicase, FBH1 [11]. Both pathways require the loading of RAD51 nucleofilaments that displace RPA at the end of the extruded arm of nascent DNA [12]. The “chicken foot” structure stabilizes the stalled fork and allows time for the resolution of replication stress. However, the extruded arm of nascent DNA is inherently susceptible to nucleolytic processing by specific nucleases [1,2,3,13,14,15]. The remodeling pathways mediated by the SNF2-DNA translocases and FBH1 helicase are susceptible to attack from different nucleases [16]. The SNF2-DNA translocase-mediated pathway is susceptible to MRE11 and EXO1 nuclease attack, whereas the FBH1 helicase pathway is sensitive to DNA2 processing [14,15,17,18,19,20]. To prevent destabilization of the “chicken foot” structure by nuclease activity, these two remodeling pathways recruit different replication fork protection factors. The SNF2-DNA translocases stimulate the recruitment of breast cancer proteins BRCA1 and BRCA2, together with the Fanconi anemia (FA) complex, FANCD2-FANCI, and BRCA interacting protein ABRO1, to protect against MRE11 and EXO1 nucleases [21,22,23,24,25]. In contrast, during FBH1 helicase-mediated fork protection from DNA2, nuclease activity is ensured through the recruitment of the FA factor FANCA and the P53 binding protein 53BP1, as well as the Von Hippel–Lindau (VHL) tumor suppressor and Biorientation Defect 1-like (BOD1L) proteins [25,26,27,28,29]. Although there are likely structural differences between the two DNA replication fork regression pathways, these are yet to be elucidated. Nevertheless, once the nucleotide levels rise, the “chicken foot” structure is unwound and the nascent fork structure is restored to allow for the resumption of DNA synthesis without additional processing (Figure 1). On the other hand, a prolonged nucleotide deficit will lead to the “chicken foot” structure being processed by the SLX4–MUS81–EME1 structure-specific endonuclease complex [16,30,31]. This process has been termed a form of replication fork collapse and leads to the formation of a double-strand break (DSB), which is repaired via homologous recombination [14,32].

Also crucial in the replication stress response is the enactment of large-scale structural changes promoting faithful genome duplication. Structural maintenance of chromosomes (SMC) complexes cohesin and condensin have well-defined roles in sister chromatid cohesion, chromosome compaction, and chromosome assembly, which are unsurprisingly essential for DNA replication, allowing for the spatial reorganization of chromatin, the relief of superhelical tension, and the reestablishment of sister chromatid cohesion upon genome duplication [33,34,35,36,37,38,39,40]. The functions of the third SMC complex family member, SMC5/6, are more enigmatic, but are clearly important for DNA replication fidelity [41,42,43]. In mammalian cells, SMC5/6 ensures the stability of DNA replication forks, the completion of DNA replication, and faithful chromosome segregation [44,45,46]. Studies using budding and fission yeasts have shown that Smc5/6 accumulates at loci in the genome that are prone to replication stress, and that play a role in fork stabilization and DNA damage repair via homologous recombination (HR) mechanisms [47,48,49,50,51,52,53,54]. These results indicate an essential role of the SMC5/6 complex in processing DNA replication intermediates.

In vitro studies of the DNA binding properties of isolated human SMC5/6 components have revealed a strong preference of the SMC5 and SMC6 monomers for binding single-stranded DNA (ssDNA) [55,56,57]. However, heterodimerization significantly increases the affinity of the complex for double-stranded DNA (dsDNA) substrates [56]. It was later discovered that certain residues within the Smc5/6 hinge domain, termed the ‘latch’ and ‘hub’, are key points of interaction with ssDNA, and these ssDNA interactions, while not required for the chromatin association of SMC5/6, have been implicated in the replication stress response [58]. Recent revelations into the structure and DNA-binding properties of SMC5/6 have illuminated a potential role as a molecular machine, facilitating the entrapment, compaction, and stabilization of replication-associated DNA tertiary structures [59,60]. Using correlative single-molecule fluorescence and force microscopy, it has been demonstrated that SMC5/6 dynamically associates with dsDNA while stably binding to ss-dsDNA junctions [61].

Other studies have suggested that SMC5/6 functions as a hub of protein–protein interaction by way of its coiled-coil arms which, in notable contrast to the other SMC complexes, contain extensive binding sites for other proteins as revealed by cross-linking MS [62]. Indeed, SMC5/6 physically interacts with replication-fork-associated proteins like the FANCD2-I subcomplex and functions epistatically with FA core complex factors FANCC, FANCM, and FANCJ in the repair of various replication-associated DNA lesions in DT40 and HeLa cells [63]. It was postulated that SMC5/6 operates downstream of fork stabilization by FANCD2-I, though this warrants further investigation. Proximity labeling studies in human cells have also identified SMC5/6 as a putative interaction partner of 53BP1 and BRCA1 [64], which function in the FBH1 helicase- and SNF2-DNA translocase-mediated replication fork protection pathways, respectively [25]. ChIP-seq analysis has demonstrated that BRCA1 and SMC5/6 occupy many of the same genomic loci upon treatment with HU [65]. Therefore, it is foreseeable that SMC5/6 could promote fork protection by regulating the activity of 53BP1 and BRCA1.

For DNA structures to be maintained and efficiently transition from one state to another, it is essential that protein stabilization and turnover are controlled. It has been demonstrated that cullin ring ligases (CRLs) are involved in controlling these processes. CRLs localize to actively replicating regions of DNA upon replication stress induction [66,67,68,69]. In particular, CRL4 has been shown to be required for the retention of replication fork protection factors and has been functionally connected with the FA pathway, stimulating the fork protection activity of FANCD2 [70,71,72]. However, the precise function of CRL-mediated protein turnover in promoting replication fork protection is largely unelucidated. The E3 ubiquitin ligase activity of CRLs is negatively regulated by the COP9 signalosome (CSN), a cullin deneddylase, components of which are also found to be constitutively associated with the active replication fork [69]. Taken together, these findings reflect the importance of regulated protein turnover at the replisome, maintaining replication fork stability through a yet unknown mechanism.

Intriguingly, physical interactions between the multiple components of the SMC5/6 complex and the CSN were reported [73]. It was also shown that the chromatin localization of SMC5/6 to sites of DNA damage is enhanced when the activity of the CSN is inhibited, suggesting that these two complexes are functionally and physically linked, and their molecular relationship appears to be an antagonistic one [73]. SMC5/6 has also been connected to CRL4 in its emerging capacity as a viral restriction factor. The hepatitis B virus protein HBx forms a complex with CRL4 to target components of SMC5/6 for proteasomal degradation [74,75,76]. However, it has been demonstrated that even in the absence of viral infection and HBx protein, SMC5/6 interacts with CRL4, and its levels show a dependence on CRL activity [77]. Therefore, it is likely that a broader role for the apparent interaction between SMC5/6, the CSN, and CRL4 exists.

In this study, we investigate the molecular consequences of SMC5/6 loss at the replication fork in mouse embryonic stem cells (mESCs), employing the auxin-inducible degron (AID) system to deplete SMC5 acutely and reversibly in the defined cellular contexts of replication fork stall and restart. We demonstrate that SMC5/6 is required to protect a stalled replication fork from MRE11 nuclease activity by stabilizing the localization of DNA replication fork protection factors. Further, we show evidence that the SMC5/6 complex is preventing the CSN from deactivating CRL functions that are required for stabilizing a stalled replication fork and subsequently ensure replication restart.

## 2. Results

### 2.1. SMC5/6 Is Required for Replication Fork Restart following Exogenous Replication Blockade

**Figure 2:**
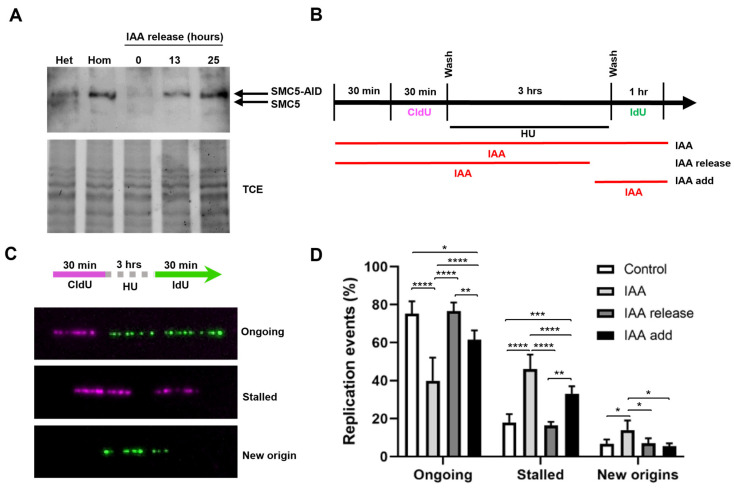
SMC5/6 is required for replication fork restart. (**A**) Western blot analysis of SMC5-AID protein levels prior to IAA treatment and after 3 h of IAA (100 µM) treatment followed by removal of IAA for 0 h, 13 h, and 25 h in *Smc5-AID* heterozygous (Het) and homozygous (Hom) mESCs. The upper band represents SMC5-AID protein, while the lower band represents endogenous SMC5 protein, present only in *Smc5-AID* heterozygous mESCs. 2,2,2-Trichloroethanol (TCE) was incorporated in the gel to visualize total loaded protein (lower panel). (**B**) Schematic of CldU (250 µM) and IdU (250 µM) labeling, HU (2 mM) treatment, and three different IAA treatment conditions. IAA was either present for the entire time course (IAA), washed out (IAA release), or added (IAA add) 30 min prior to release from HU. (**C**) Representative images of DNA fibers depicting ongoing and stalled replication forks, and newly fired origins. (**D**) Quantification of replication event frequency in control, IAA, IAA release, and IAA add conditions. Data represent mean ± S.E.M. (control condition: n = 307 fibers; IAA condition: n = 491 fibers; IAA release condition: n = 430 fibers; IAA add condition: n = 191 fibers; 3 experiments were performed for each condition). Pearson’s chi-squared test was used with Yates’ continuity correction. * *p* < 0.05, ** *p* < 0.005, *** *p* < 0.0005, **** *p* < 0.0001. See Supplementary Table S2 for *p*-values and statistics.

To investigate the role of SMC5/6 during the response to replication stress, we utilized mESCs harboring the AID system (homozygous for *Smc5-AID* and the *OsTir1* transgene) for an acute and reversible depletion of SMC5 protein (Figure 2A). In agreement with previous studies [44,78], SMC5 was fully depleted after 1 h of indole-3-acetic acid (IAA) administration, and following the removal of IAA, SMC5 protein levels gradually recovered and were restored to basal levels (Figure 2A, Supplementary Figure S1A).

Having established the utility of the AID system for the rapid and reversible depletion of SMC5, we sought to determine the requirement for SMC5/6 during replication fork restart following an exogenous replication blockade. We performed DNA fiber assays in which cells were pulsed with the thymidine analog CldU, followed by treatment with the ribonucleotide reductase inhibitor hydroxyurea (HU) to induce replication fork stalling, and subsequent pulse labeling with the nucleotide analog IdU (Figure 2B,C, Supplementary Figure S1B,C). We found that the frequency of ongoing replication forks after the removal of HU in SMC5-depleted mESCs was 1.9-fold lower than that of non-IAA-treated control cells (Figure 2D). Moreover, SMC5 depletion led to a 2.6-fold increase in rates of fork stalling and a 2.0-fold increase in new origin firing. Strikingly, the withdrawal of IAA for 1 h, allowing for the return of the SMC5 protein, restored replication fork restart rates to levels nearly indistinguishable from the control. These observations were recapitulated when the replication block was mediated by the DNA polymerase inhibitor aphidicolin (Supplementary Figure S1D,E). Overall, our results indicate that SMC5/6 is required during recovery from the replication stall for efficient replication fork restart. Intriguingly, when SMC5 was depleted only during recovery from HU treatment, fork restart was slightly impaired, but this defect was markedly less severe than that of SMC5 depletion during the entire experimental workflow (Figure 2D). This suggests that the primary function of the SMC5/6 complex is to maintain restart-competent replication forks following a replication fork stall.

### 2.2. SMC5/6 Promotes Localization of Fork Protection Factors to Stalled Replication Forks

**Figure 3:**
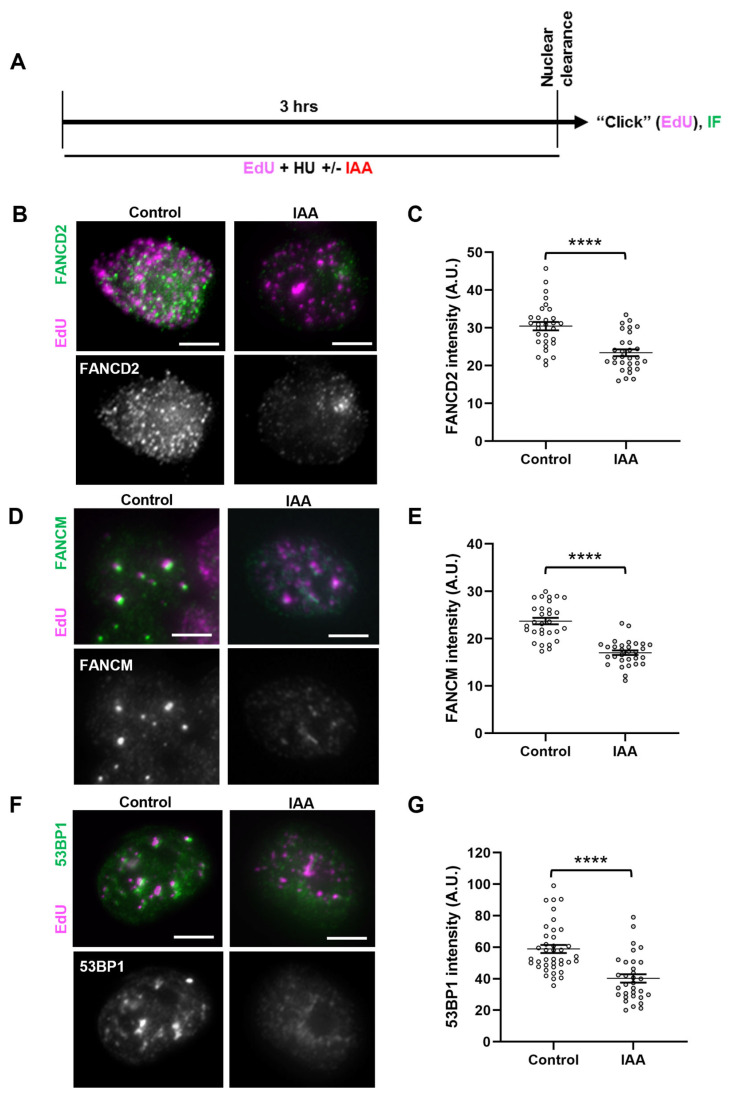
SMC5/6 promotes localization of fork protection factors to stalled replication forks. (**A**) Schematic of EdU labeling and HU and IAA treatment for immunofluorescence (IF) analysis following nuclear clearance preparation of mESCs. (**B**) Representative nuclear clearance images of FANCD2 (green) staining and EdU (magenta) incorporation in control and IAA-treated *Smc5-AID* mESCs. Scale bar: 5 µm. (**C**) Quantification of average FANCD2 focus intensity per nucleus in control and IAA-treated *Smc5-AID* mESCs following nuclear clearance preparation. Data represent mean ± S.E.M. (control condition: n = 40 cells; IAA condition: n = 41 cells; 3 experiments were performed for each condition). Unpaired two-tailed Mann–Whitney test; **** *p* < 0.0001. (**D**) Representative nuclear clearance images of FANCM (green) staining and EdU (magenta) incorporation in control and IAA-treated *Smc5-AID* mESCs. Scale bar: 5 µm. (**E**) Quantification of average FANCM focus intensity per nucleus in control and IAA-treated *Smc5-AID* mESCs following nuclear clearance preparation. Data represent mean ± S.E.M. (control condition: n = 41 cells; IAA condition: n = 43 cells; 3 experiments were performed for each condition). Unpaired two-tailed Mann–Whitney test; **** *p* < 0.0001. (**F**) Representative nuclear clearance images of 53BP1 (green) staining and EdU (magenta) incorporation in control and IAA-treated *Smc5-AID* mESCs. Scale bar: 5 µm. (**G**) Quantification of average 53BP1 focus intensity per nucleus in control and IAA-treated *Smc5-AID* mESCs following nuclear clearance preparation. Data represent mean ± S.E.M. (control condition: n = 43 cells; IAA condition: n = 45 cells; 3 experiments were performed for each condition). Unpaired two-tailed Mann–Whitney test; **** *p* < 0.0001. See Supplementary Table S2 for *p*-values and statistics.

Given the fork instability phenotype observed upon SMC5 depletion, we sought to determine whether certain replication fork protection pathways were perturbed in SMC5-depleted cells. By the assessment of DNA resection at a stalled replication fork, it was shown that there are two main replication fork protection pathways that act downstream of RAD51: one pathway that relies on the FA protein FANCD2, and the other on 53BP1 [25]. Therefore, we assessed the localization of FANCD2 and 53BP1 fork protection factors to stalled replication forks in SMC5-depleted mESCs via an immunofluorescence microscopy analysis of replication-fork-associated proteins in HU-treated, EdU-labeled mESCs, using the nuclear clearance approach (Figure 3A) [79,80]. This approach includes a brief incubation of mESCs in nonionic detergent to remove all non-chromatin-associated proteins in the cell. The remaining protein signals detect mark sites that are bound to the chromatin. We observed that while the number of foci for FANCD2 and 53BP1 did not change between the SMC5-depleted and control groups, the intensity of their signals significantly changed when SMC5 was depleted. In SMC5-depleted mESCs, we observed a 1.3-fold decrease in levels of EdU-associated FANCD2 (Figure 3B,C). Previous studies have shown that the recruitment of FANCD2 to the replication fork is required for the retention and fork processing activity of the fork remodeler FANCM [81,82]. Consistent with this, we also observed a 1.4-fold decrease in levels of EdU-associated FANCM upon SMC5 depletion, with no reduction in the number of foci per cell (Figure 3D,E). We also recorded a 1.4-fold reduction in 53BP1 levels that colocalized with EdU in SMC5-depleted mESCs (Figure 3F,G). In addition to FANCM, FANCD2, and 53BP1, we assessed SMARCAL1, BRCA1, BRCA2, and CtIP. However, we did not observe discernable differences between SMC5-depleted and control conditions for these factors using the nuclear clearance technique. Thus, the SMC5/6 complex is important for physical fork protection by both the FANCD2 and 53BP1 pathways and promotes the recruitment and/or retention of FANCM. These data demonstrate the multifaceted role of the SMC5/6 complex in mounting an appropriate replication stress response. Given that FANCM has been shown to regulate homologous-recombination-mediated repair mechanisms at stalled replication forks in mESCs [83], it is possible that the absence of SMC5/6 leads to fork collapse and an inability to undergo error-free homologous recombinational repair, a theory that is supported by prior work using budding and fission yeast and mammalian cells [42,43].

### 2.3. Loss of SMC5/6 Leads to MRE11-Dependent Replication Fork Destabilization

During replication fork stalling, ssDNA is coated by RPA, which is then displaced by RAD51 [84]. RAD51 mediates fork regression in conjunction with several DNA translocases and helicases, including FANCM [3,32]. The extruded arm of the nascent DNA arm of the “chicken foot” structure must be stabilized and protected by factors such as RAD51, BRCA2, and FANCD2-I (among many others) to prevent uncontrolled processing by nucleases [1,2]. When fork protection is lost, excess resection, initiated by MRE11 nuclease, ultimately results in the cleavage of the regressed fork “chicken foot” structure [1,14,32]. This process, termed replication fork collapse, generates a single-ended DSB which must resume DNA synthesis via HR-directed methods [14,32]. The maintenance of fork protection is therefore critically important for preventing the excess processing of stalled replication forks, fork collapse, and unscheduled recombination.

**Figure 4:**
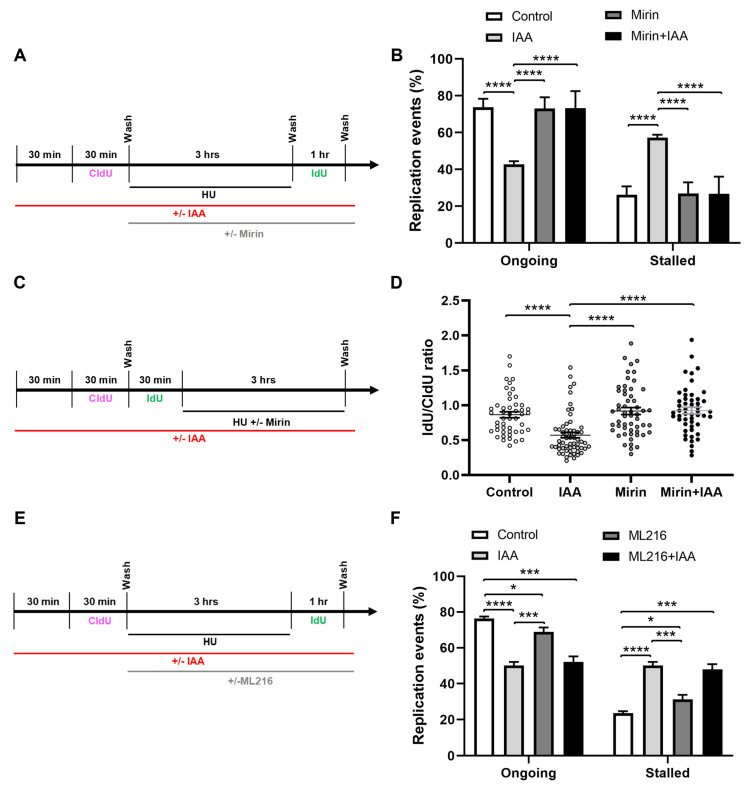
Fork restart defect upon SMC5/6 loss is underpinned by MRE11-dependent replication fork destabilization. (**A**) Schematic of CldU and IdU labeling and HU, IAA, and mirin treatment. (**B**) Quantification of replication event frequency in control and IAA-treated mESCs, with or without mirin treatment. Data represent mean ± S.E.M. (control condition: n = 459 fibers; mirin condition: n = 390 fibers; IAA condition: n = 489 fibers; mirin+IAA condition: n = 512 fibers; 3 experiments were performed for each condition). Pearson’s chi-squared test with Yates’ continuity correction; **** *p* < 0.0001. (**C**) Schematic of CldU and IdU labeling and HU, IAA, and mirin treatment. (**D**) Quantification of CldU to IdU track length ratio in dual-labeled fibers in control and IAA-treated mESCs, with or without mirin treatment. Data represent mean ± S.E.M. (control condition: n = 48 fibers; mirin condition: n = 55 fibers; IAA condition: n = 59 fibers; mirin+IAA condition: n = 51 fibers; 3 experiments were performed for each condition). Unpaired two-tailed Mann–Whitney test; **** *p* < 0.0001. (**E**) Schematic of CldU and IdU labeling and HU, IAA, and ML216 treatment. (**F**) Quantification of replication event frequency in control and IAA-treated mESCs, with or without ML216 treatment. Data represent mean ± S.E.M. (control condition: n = 383 fibers; ML216 condition: n = 414 fibers; IAA condition: n = 434 fibers; ML216+IAA condition: n = 535 fibers; 2 experiments were performed for each condition). Pearson’s chi-squared test with Yates’ continuity correction; * *p* < 0.05, *** *p* < 0.0005, **** *p* < 0.0001. See Supplementary Table S2 for *p*-values and statistics.

We hypothesized that SMC5/6 might play a role in the prevention of the MRE11-dependent resection of stalled replication forks. To assess whether MRE11 nuclease activity was responsible for the replication fork restart defect of SMC5-depleted mESCs, we performed DNA fiber assays in the presence of HU and mirin, an inhibitor of MRE11 3′-5′ exonuclease activity (Figure 4A) [85]. In agreement with previous studies, treatment with mirin alone did not significantly impair fork restart ability in mESCs [14,86]. Strikingly, we observed that the fork restart defect of IAA-treated mESCs was rescued by concurrent treatment with mirin (Figure 4B). This phenotype was recapitulated when the replication block was mediated by the DNA polymerase inhibitor aphidicolin (Supplementary Figure S2A,B). Consistent with these observations, treatment with PFM01, an inhibitor of MRE11 endonuclease activity [87], also rescued the fork restart defect of SMC5-depleted mESCs (Supplementary Figure S2C,D). This observation may indicate that MRE11 endonuclease activity is required to stimulate exonuclease activity and efficient end resection, as previously reported [88,89]. We also inhibited the 5′-3′ exonuclease activity of DNA2 in similar DNA fiber assays (Supplementary Figure S2E). However, DNA2 inhibition did not restore fork protection in SMC5-depleted mESCs (Supplementary Figure S2F). This aligns with what has been observed when BRCA1 or BRCA2 are depleted, which are rescued by MRE11 depletion or inhibition but not DNA2 depletion or inhibition [14,90]. However, it is also possible that these observations are due to redundancy with other exonucleases, including with EXO1 [91].

Next, we sought to determine whether SMC5 depletion led to an increased MRE11-dependent degradation of nascent DNA at stalled forks. We performed DNA fiber assays in which mESCs were sequentially pulsed with CldU and IdU, followed by treatment with HU, in the presence or absence of IAA and mirin (Figure 4C). We found that the IdU-containing track length, measured in unidirectional replication forks with both CldU and IdU incorporation, was 1.4-fold shorter in SMC5-depleted mESCs compared to the control (Figure 4D). In contrast, the track lengths were restored to levels similar to the control upon treatment with mirin. Overall, these data suggest that SMC5/6 prevents an excessive MRE11-dependent resection of stalled replication forks, and that this activity is essential to preserve replication fork restart competence.

### 2.4. Loss of SMC5/6 Causes More Severe Fork Restart Defect Than Inhibition of BLM Helicase

Bloom’s syndrome (BLM) helicase is known to stabilize stalled replication forks and prevent replication fork collapse [92,93]. The function of the SMC5/6 complex has been repeatedly linked with the BLM helicase, particularly in budding yeast studies, where the SMC5/6 complex has been shown to interact and regulate BLM function [48,49,54,94,95]. Therefore, we sought to determine the contribution of BLM following a DNA replication fork stall (Figure 4E). We performed DNA fiber assays using ML216, a selective inhibitor of BLM helicase DNA unwinding activity [96]. We found that SMC5 depletion resulted in a fork restart defect 1.3-fold more severe than that of BLM inhibition, while the inhibition of BLM helicase concurrently with SMC5 depletion did not significantly exacerbate the fork restart defect caused by SMC5 depletion [50] (Figure 4D). These results suggest that SMC5/6 and BLM may be epistatic and function in the same pathway. Additionally, the data imply that SMC5/6 has additional roles apart from promoting the BLM helicase functions that are required for the efficient restart of stalled replication forks. This observation is consistent with prior observations that suggest there are roles for the SMC5/6 complex that are independent of the BLM helicase [49,97,98].

### 2.5. SMC5/6 Depletion Leads to Nuclear Accumulation of CSN Components and CSN- and CRL4-Dependent Replication Fork Destabilization

Next, we sought to determine the mechanism by which SMC5/6 stabilizes replication forks and promotes fork restart. In recent years, the SMC5/6 complex has been shown to act as a restriction factor for several viruses, preventing their replication [74,75,99,100,101,102,103,104]. The Hepatitis B virus protein X (HBx), which is required for viral replication and transcription, interacts with the cullin-ring ligase complex CRL4 and SMC5/6 to promote viral replication within the cell [74,75,102]. Murphy and colleagues propose that HBx acts as a link between CRL4 and SMC5/6 complexes, targeting SMC5/6 components for CRL4-mediated proteasomal degradation [75]. However, recent work has demonstrated that even in the absence of hepatitis virus infection, SMC6 interacts with CRL4 and, upon pevonedistat-mediated CRL4 inhibition, SMC5/6 levels increase [77].

CRL4 is an essential regulator of DNA replication, transcription, and repair, as well as cell cycle progression [105,106,107,108,109,110,111,112]. The activity of CUL4 and other cullins requires NEDD8, a small ubiquitin-like protein that is covalently conjugated to cullins and facilitates the recruitment of E2 for the ubiquitination of CRL substrates. CRL activity is modulated by the CSN, a deneddylase that removes NEDD8 from the cullin component of CRLs, rendering the CRL enzymatically inactive [113,114,115]. The CSN comprises nine distinct subunits (CSN1-9), of which CSN5 is the catalytic subunit directly responsible for cullin deneddylation [113,114,115]. Intriguingly, multiple components of the SMC5/6 complex have been shown to physically interact with components of the CSN, suggesting that there may be a functional interplay between these two complexes [73]. Considering the established roles of CRL4 and its accessory factors in DNA replication, it can be postulated that SMC5/6 modulates the activity of CRL4 at the replication fork via regulation of the CSN.

**Figure 5:**
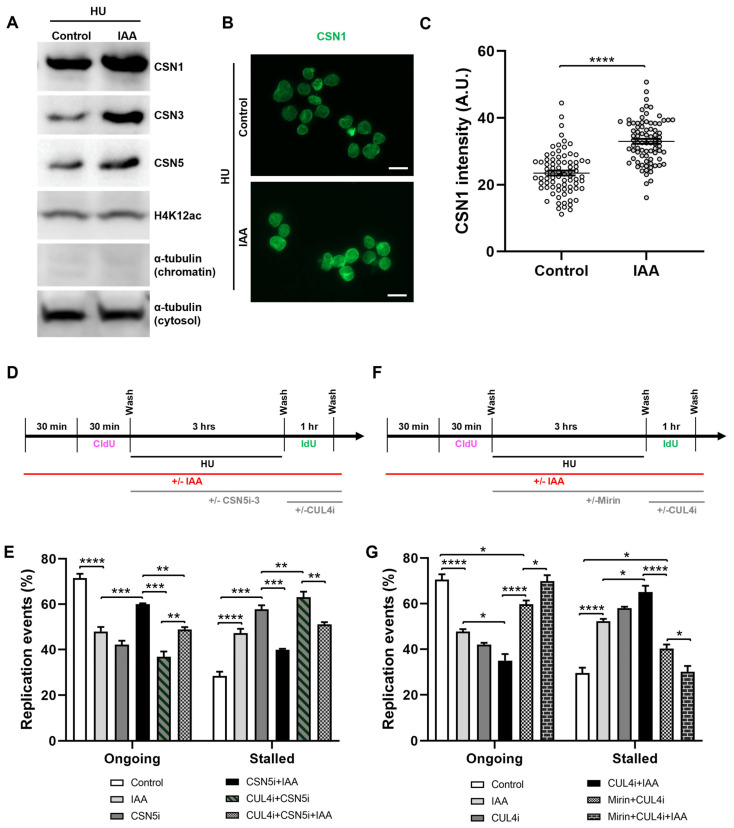
SMC5/6 depletion leads to nuclear accumulation of CSN components and CSN- and CRL4-dependent replication fork destabilization. (**A**) Western blot analysis of CSN1, CSN3, and CSN5 protein levels in chromatin fraction of control and IAA-treated mESCs after 3 h of HU treatment. Alpha-tubulin (chromatin and cytosol fractions) and H4K12ac were used as controls. 2,2,2-Trichloroethanol (TCE) was incorporated in the gel to visualize total loaded protein (lowest panel). (**B**) Representative nuclear clearance images of CSN1 (green) staining in control and IAA-treated *Smc5-AID* mESCs. Scale bar: 20 µm. (**C**) Quantification of average CSN1 intensity per nucleus in control and IAA-treated *Smc5-AID* mESCs following nuclear clearance preparation. Data represent mean ± S.E.M. (control condition: n = 78 cells; IAA condition: n = 81 cells). Unpaired two-tailed Mann–Whitney test; **** *p* < 0.0001. (**D**) Schematic of CldU and IdU labeling and HU, IAA, CSN5i-3 (2.5 µM) (CSNi), and compound 33-11 (25 µM) (CUL4i) treatment. (**E**) Quantification of replication event frequency in control and IAA-treated mESCs, with or without CSN5i-3 and CUL4i treatment. Data represent mean ± S.E.M. (control condition: n = 309 fibers; IAA condition: n = 456 fibers; CSNi condition: n = 237 fibers; CSNi+IAA condition: n = 418 fibers; CSNi+CUL4i condition: n = 275 fibers; CSNi+CUL4i+IAA condition: n = 261 fibers). Three experiments were performed for each condition. Pearson’s chi-squared test with Yates’ continuity correction; ** *p* < 0.005, *** *p* < 0.0005, **** *p* < 0.0001. Significance is not shown for all groups. See Supplementary Table S2 for *p*-values and statistics. (**F**) Schematic of CldU and IdU labeling and HU, IAA, mirin (Mir), and compound 33-11 (CUL4i) treatment. (**G**) Quantification of replication event frequency in control and IAA-treated mESCs, with or without mirin and CUL4i treatment. Data represent mean ± S.E.M. (control condition: n = 367 fibers; IAA condition: n = 401 fibers; CUL4i condition: n = 355 fibers; CUL4i+IAA condition: n = 280 fibers; CUL4i+Mirin condition: n = 322 fibers; CUL4i+Mirin+IAA condition: n = 212 fibers). Three experiments were performed for each condition. Pearson’s chi-squared test with Yates’ continuity correction; * *p* < 0.05, **** *p* < 0.0001. Significance not shown for all groups. See Supplementary Table S2 for *p*-values and statistics.

To assess a potential interaction between SMC5/6 and the CSN in the context of DNA replication, we performed Western blot analyses of chromatin-associated CSN components in fractionated mESCs. We observed that levels of CSN1, CSN3, and CSN5 significantly increased upon IAA treatment in the context of HU-mediated replication stress (Figure 5A, Supplementary Figure S3A). To complement the Western blot assessments, we performed immunofluorescence analyses of chromatin-associated CSN1 in HU-treated mESCs, using a nuclear clearance approach for the removal of cytoplasmic and nucleoplasmic signals and an improved visualization of chromatin-bound proteins. In accordance with Western blot analyses, we recorded an overall 1.4-fold increase in the intensity of CSN1 in IAA-treated mESCs compared to the control cells (Figure 5B,C, Supplementary Figure S3B,C).

To assess the role of CSN regulation by SMC5/6 in replication fork restart, we performed DNA fiber assays in the presence or absence of IAA and a CSN5 inhibitor (CSN5i-3) (Figure 5D). Strikingly, we found that CSN5 inhibition in SMC5-depleted mESCs partially restored fork restart ability to that of the control, with fork restart efficiency increasing 1.4-fold compared to CSN5i-3-treated mESCs. We posited that the observed rescue of fork restart by CSN inhibition might reflect a role of SMC5/6 in negatively regulating the CSN, thereby promoting the functions of CRLs. Due to the established links between SMC5/6 and CRL4, we next assessed the role of CUL4 in promoting fork restart in SMC5-depleted mESCs. We performed DNA fiber assays in the presence of a CUL4 inhibitor (compound 33-11) following HU washout, with or without CSN inhibition and SMC5 depletion. The addition of CUL4 inhibitor alone had markedly negative effects on fork restart, with a 1.9-fold decrease in fork restart efficiency compared to the control cells, reflective of the established roles of CRL4 in promoting replication fork restart and stability (Figure 5E). Moreover, the treatment of SMC5-depleted, CSN5-inhibited mESCs with the CRL4 inhibitor abolished the rescue of fork restart rates observed with the inhibition of CSN5 (Figure 5E). Therefore, we concluded that SMC5/6 negatively regulates CSN activity at the replication fork to promote fork restart and that this fork restart is dependent on CRL4 activity. We propose that the regulation of CSN by SMC5/6 is important for promoting the functions of CRL4 at the replication fork.

### 2.6. SMC5/6 and CRL4 Prevent MRE11-Dependent Replication Fork Instability

Given our observation that CSN inhibition rescued fork restart defects associated with SMC5 depletion and that this rescue was dependent on CRL4 activity, we wondered whether SMC5/6 may facilitate the fork protection functions of CRL4. We hypothesized that the depletion of SMC5, causing disruption to CRL4 functions, would lead to subsequent MRE11-mediated fork instability. To determine whether the fork restart defect in the absence of SMC5/6 and CRL4 activity was MRE11-dependent, we performed DNA fiber assays in the presence or absence of IAA, mirin, and the CUL4 inhibitor (Figure 5F). The CUL4 inhibitor was added when HU was removed to specifically assess the consequences of CRL4 inhibition when DNA replication is able to restart. We found that the addition of mirin to mESCs treated with compound 33-11 rescued fork restart rate to levels almost to that of the control condition, with a modest decrease of 1.2-fold (Figure 5G). Moreover, the addition of mirin to mESCs treated with IAA, and subsequently the CUL4 inhibitor following HU washout, led to the rescue of fork restart ability to levels similar to the control (Figure 5G). Overall, these data suggest that an excess MRE11-dependent degradation of stalled replication forks is responsible for fork instability in the absence of CRL4 and SMC5/6 activity. Due to the various functions known of CRL4 during DNA replication and repair [116], we acknowledge that CLR4 may be working in multiple pathways during DNA replication fork recovery, independent to that of SMC5/6. For instance, CRL4, together with its substrate receptor DCAF14, protects nascent DNA from MRE11 and DNA2 nucleases [70]. On the other hand, we showed that replication fork protection is not restored upon DNA2 inhibition in SMC5-depleted mESCs (Supplementary Figure S2F).

### 2.7. SMC5/6 Promotes Localization of Fork Protection Factors to Stalled Replication Forks by Negatively Modulating CSN

**Figure 6:**
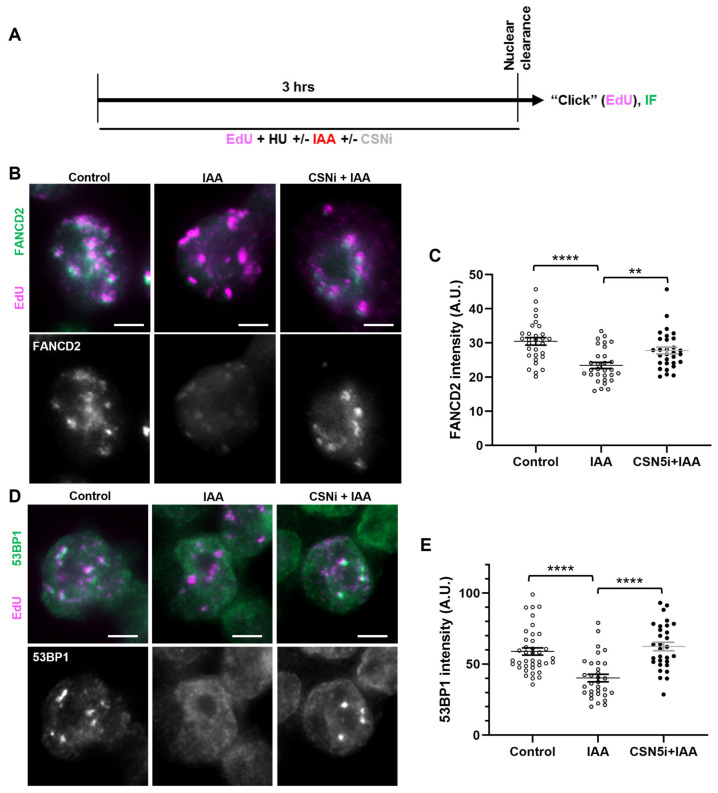
SMC5/6 promotes localization of fork protection factors to stalled replication forks by negatively modulating CSN. (**A**) Schematic of EdU labeling and HU, IAA, and CSN5i-3 treatment for immunofluorescence (IF) analysis with nuclear clearance. (**B**) Representative nuclear clearance images of FANCD2 (green) staining and EdU (magenta) incorporation in control, IAA-, and CSN5i-3-treated *Smc5-AID* mESCs. Scale bar: 5 µm. (**C**) Quantification of average FANCD2 focus intensity per nucleus in control, IAA-, and CSN5i-3-treated *Smc5-AID* mESCs following nuclear clearance preparation. Data represent mean ± S.E.M. (control condition: n = 40 cells; IAA condition: n = 41 cells; CSNi+IAA condition: n = 47 cells). Three experiments were performed for each condition. Unpaired two-tailed Mann–Whitney test; ** *p* < 0.0005, **** *p* < 0.0001. See Supplementary Table S2 for details. (**D**) Representative nuclear clearance images of 53BP1 (green) staining and EdU (magenta) incorporation in control, IAA-, and CSN5i-3-treated *Smc5-AID* mESCs. Scale bar: 5 µm. (**E**) Quantification of average 53BP1 focus intensity per nucleus in control, IAA-, and CSN5i-3-treated *Smc5-AID* mESCs following nuclear clearance preparation. Data represent mean ± S.E.M. (control condition: n = 43 cells; IAA condition: n = 45 cells; CSNi+IAA condition: n = 50 cells). Three experiments were performed for each condition. Unpaired two-tailed Mann–Whitney test; **** *p* < 0.0001. See Supplementary Table S2 for *p*-values and statistics.

We next assessed the localization of the fork protection factors FANCD2 and 53BP1 to the stalled replication forks in SMC5-depleted mESCs with CSN5 inhibition. We performed immunofluorescence assessments of replication-fork-associated proteins in HU-treated, EdU-labeled mESCs, using the nuclear clearance approach (Figure 6A). Strikingly, in SMC5-depleted mESCs, the addition of the CSN5 inhibitor restored the levels of EdU-associated FANCD2 and 53BP1 in SMC5-depleted mESCs to levels similar to the control (Figure 6B–E). Thus, we have shown evidence to suggest that SMC5/6 promotes the recruitment of replication fork protection factors FANCD2 and 53BP1 to stalled replication forks by negatively modulating the activity of the CSN.

## 3. Discussion

### 3.1. SMC5/6 Promotes Replication Fork Stability and Restart

In this study, we have directly examined replication fork dynamics upon acute SMC5 depletion using fine-tuned auxin-mediated protein degradation in ESCs. We have demonstrated a requirement for SMC5/6 function to enable the efficient restart of DNA replication forks following replication stalling. This requirement is underpinned by the role of SMC5/6 in preventing the MRE11-mediated nucleolytic processing of stalled replication forks, thereby promoting replication fork stability and preventing replication fork collapse. Our observations are consistent with numerous studies on yeast describing the accumulation of recombination intermediates stemming from fork collapse events upon Smc5/6 loss [41,42,43,117,118]. Our findings highlight a direct role of SMC5/6 in stabilizing stalled replication forks and underscore the importance of this function to ensure the progression of DNA replication. It is not surprising that recent studies assessing or modeling mutations of SMC5/6 components seen in humans all demonstrate a degree of genome instability linked with an inability to complete DNA replication prior to chromosome segregation [119,120,121,122].

**Figure 7:**
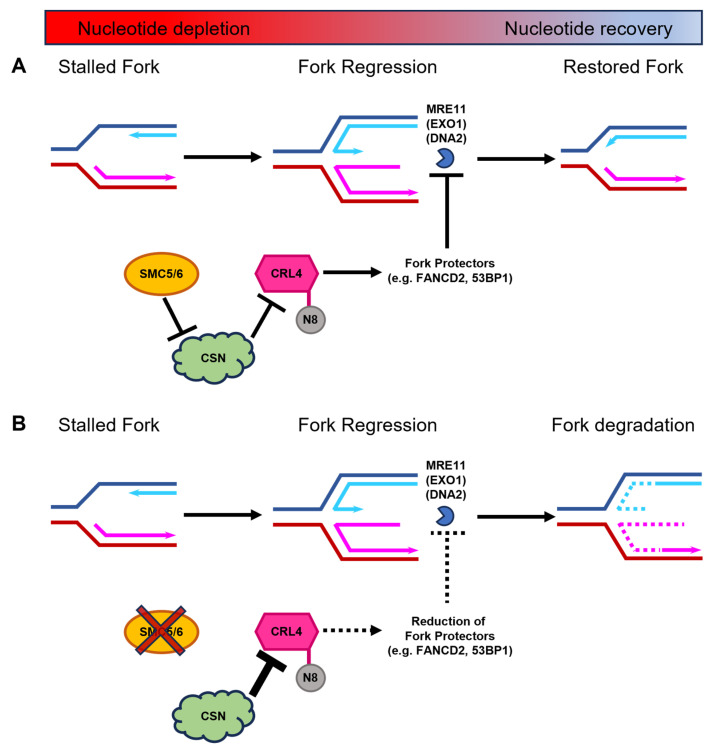
Proposed mechanism for the SMC5/6 complex during DNA replication fork stalling and stabilization during nucleotide depletion. (**A**) During nucleotide deficiency, regressed DNA replication forks are protected by replication fork protectors, including FANCD2 and 53BP1. These proteins rely on the ubiquitination function of CRL4. CRLs are only active when conjugated to the small ubiquitin-like protein NEDD8 (N8). CSN-mediated deneddylation of CRLs renders them enzymatically inactive. We propose that SMC5/6, through mechanism that is yet to be fully elucidated, negatively regulates CSN to ensure that CRL4 remains active during DNA replication fork stalling to safeguard replication forks against nuclease-mediated degradation. Thus, regressed forks can subsequently undergo restoration of the DNA replication process when nucleotide levels rise. (**B**) In the absence of the SMC5/6 complex (depicted by red cross out of SMC5/6), CSN levels increase, which enhances their capacity to deneddylate CRL4 (depicted by larger inhibition arrow). Thus, the diminished levels of active CRL4 result in decreased recruitment of key replication fork protection factors (depicted by dashed lines), exposing the stalled DNA replication fork to degradation by MRE11 and potentially other nucleases, such as EXO1 and DNA2. Destabilization of the DNA replication fork leads to an inability to restart replication following replenishment of available nucleotides.

The depletion of SMC5 only during recovery from replication stall does not cause a severe fork restart defect, compared to the depletion of SMC5 during both replication stall and recovery. This suggests that the functions of SMC5/6 specifically during fork stall are critical to avert replication fork collapse. We propose that during a replication fork stall, SMC5/6 enables replication fork protection by negatively modulating the activity of CSN and promoting the activity of CRLs. This function is essential for maintaining the presence of replication fork protection factors, the suppression of MRE11-mediated fork degradation, and the prevention of fork collapse (Figure 7).

### 3.2. SMC5/6 Promotes the Functions of 53BP1 and the FA Pathway at Stalled Replication Forks

In DT40 cells, it was shown that SMC5 acts in conjunction with FANCM when exposed to cisplatin, which primarily causes the formation of intra-strand crosslinks that block high-fidelity DNA polymerases [63,123]. Furthermore, components of the SMC5/6 complex interact with the FANCD2-FANCI complex in human cells [63]. Our study showed that the depletion of SMC5/6 results in a reduced localization of FANCM and FANCD2 to stalled replication forks. In contrast, other studies have reported that the depletion of SMC5/6 does not affect FANCD2 localization following HU or aphidicolin treatment [63,98]. In fact, using the same AID system in mESCs, we showed that SMC5 depletion leads to unresolved replication intermediates that persist into mitosis (termed mitotic DNA synthesis, MiDAS), which is accompanied by increased FANCD2 foci [44]. However, the major difference between these studies and the one presented here is the exposure times within the experimental design. All prior studies assessed chronic exposures of HU or aphidicolin and the long-term depletion of SMC5/6. In contrast, our study demonstrates acute responses to DNA replication fork stall and recovery in the presence or absence of the SMC5/6 complex. Furthermore, we assessed pathways that respond to DNA replication fork stall using small-molecule inhibitors that, as with the AID system, enable the rapid perturbation of specific targets.

This study also shows that SMC5/6 is required for the stable recruitment of 53BP1 to stalled replication forks. 53BP1 operates in a replication fork protection pathway distinct from FANCD2-I [25]. Fork protection by 53BP1 controls recombinogenic processes at the replication fork [26]. Lending credence to our observations, proximity labeling studies with APEX-tagged 53BP1 have identified SMC6 as a high-confidence 53BP1 interaction partner [64]. We have previously shown that the neurodevelopmental defects caused by the mutation of *Smc5* are not further affected by co-mutation with *Trp53bp1*, suggesting that SMC5/6 and 53BP1 are potentially epistatic with one another [44].

Taken together, our studies suggest that SMC5/6 is responsible for mediating 53BP1 and FANCD2-I DNA replication fork stall-response pathways. Our data suggest that the role of the SMC5/6 is to help stabilize the DNA replication fork following fork regression. Furthermore, this function is likely mediated in conjunction with CRLs and the CSN (Figure 7). CRL4 has been functionally connected with the FA pathway, stimulating the mono-ubiquitination of FANCD2 in human cells, which is required for replication fork stabilization [70,71,124]. In addition, 53BP1 localization to sites of DNA damage is regulated by CRL4 [125]. Interestingly, it was shown that SMC5/6 localization to microlaser-induced DNA damage is not dependent on 53BP1, which suggests that 53BP1 acts downstream of the SMC5/6 complex in this context [126]. Future work should be directed towards the comprehensive analysis of stalled replication forks in the presence and absence of the SMC5/6 complex, and should determine the SMC5/6 interactome during replication fork stall and recovery.

### 3.3. SMC5/6 Mediates Replication Fork Protection and Stability by Negative Regulation of CSN

Insights from studies on the role of SMC5/6 as a viral restriction factor have directly linked its function to that of CRLs and the CSN. Multiple components of the CSN and all six components of SMC5/6 have been co-immunoprecipitated with CRL4 complexes in the context of hepatitis virus infection [74,75]. However, the interaction of SMC5/6 with CRL4 and the CSN also occurs in the endogenous cellular context, in the absence of viral infection [73,77]. The CSN has been identified as a replication-fork-associated factor and has also been implicated in repair pathway choice at DSBs, promoting end resection to initiate homologous recombinational repair [69,127]. We have presented data to suggest that, in the absence of SMC5/6, the CSN is left unregulated, leading to a loss of fork protection, increased end resection activity at stalled replication forks, and an inability to restart the replication fork (Figure 7). A preponderance of evidence suggests that most proteins canonically involved in DSB repair also play related roles at stalled replication forks [128]. Therefore, it is not surprising that in addition to its known roles in DSB repair, the CSN would also have functions in regulating replication fork progression.

Our results, taken together with existing knowledge of CSN and CRL biology, favor the notion that SMC5/6 is negatively impacting the ability of CSN to downregulate CRL activity at replication forks. (Figure 7). Protein turnover at replication forks is a highly dynamic and context-dependent process and, therefore, must be tightly controlled through multiple layers of communication between the replisome and DNA-associated factors in order to control recombinogenic processes and maintain replication fork integrity. We envision that SMC5/6 exerts a broad role in the regulation of replication fork dynamics, which are essential for preventing unscheduled recombination, mitotic catastrophe, and genome instability.

Recent structural insights have revealed that SMC5/6 acts as a DNA loop extruder that compacts DNA tertiary structures associated with DNA in a similar fashion to cohesin and condensin [129,130]. It has been posited that, rather than driving global changes to chromosome superstructures like cohesin and condensin, SMC5/6 enacts smaller-scale changes, specifically at the replication fork [59,60]. We foresee that this activity may be important for stabilizing nascent DNA structures and replication intermediates, thereby protecting them from enzymatic degradation and unscheduled recombination. The existence of extensive protein binding sites on its coiled-coil arms also differentiates SMC5/6 from the other SMC family members [62]. We have observed a disruption of multiple proteins, directing various modes of replication fork protection, upon SMC5/6 loss. We propose that SMC5/6 may act as a hub for protein–protein interactions, modulating the functions of multiple proteins at the replication fork. Perhaps therein lies the function of its E3 ubiquitin and SUMO ligase subunits, NSMCE1 and NSMCE2, respectively [42,43,131,132]. An attractive possibility is that the combined SUMOylation and ubiquitination activities of SMC5/6, together with changes in structural confirmation and protein interaction partners, provide multiple layers of regulation in response to different events at the replication fork.

We have linked the function of SMC5/6 to that of the CSN in the context of DNA replication stalled replication fork stability and restart. Little is currently known about how the CSN is regulated. Our observed functional interplay between SMC5/6 and CSN activity opens numerous avenues of further investigation, for example, determining more specifically how SMC5/6 and CSN components interact. It is known that the kleisin subunit of the SMC5/6 complex, NSMCE4A, directly interacts with CSN1 (GPS1) [73]. However, a more comprehensive assessment will be required. Perhaps clues can be drawn from the structural knowledge of the interaction between CSN and SCF complexes. These studies have revealed that in the context of cullin regulation, CSN5 and CSN4 interact with the winged helix domain and RING domain of CUL1 and its associated E3 ubiquitin ligase RBX1, respectively, in order to promote cullin deneddylation [133]. Considering that the NSMCE4A-NSMCE3-NSMCE1 subcomplex of SMC5/6 contains similarly positioned winged helix and RING domains [134,135], perhaps a similar mode of interaction occurs between CSN and SMC5/6, promoting CSN4 ubiquitination and/or occluding CSN5 access to its CRL substrates.

## 4. Materials and Methods

### 4.1. mESC Culture

The C57BL6/J (B6) mESCs used in this study were established and maintained in 2i/LIF medium, as previously described [78,136]. Briefly, mESC culture medium included 1:1 mixture of DMEM/F12 (Invitrogen, 11320-033, Waltham, MA, USA) and neurobasal medium (Invitrogen, 21103-049) with 1% N2 supplement (Invitrogen, 17502-048) and 2% B27 supplement (Invitrogen, 17504-044), 1 mM L-Glutamine (Sigma, G8540, St. Louis, MO, USA), 1% MEM non-essential amino acids (Invitrogen, 11140-050), 50 μM β-mercaptoethanol (Gibco, 21985023, Thermo Fisher Scientific, Waltham, MA, USA), 50 μg/mL BSA (Sigma, A1470), 10 ng/mL human LIF (PeproTech, 300-05, Cranbury, NJ, USA), 1 μM MEK inhibitor PD 0325901 (Cayman, 13034, Ann Arbor, MI, USA), and 3 μM GSK-3 inhibitor CHIR 99021 (Cayman, 13122-10). Cells were grown under feeder-free conditions on 0.2% gelatin and passaged every 3 days with TrypLE Express (Life Technologies, 12604013, Carlsbad, CA, USA). The B6 mESCs used in this study have been previously reported [78]. These mESCs harbored homozygous transgenes of the *Oryza sativa* TIR1 auxin receptor (TIR1) (driven by the elongation factor 1α (EF1α) promoter) that were incorporated into the *H11* locus. These mESCs also expressed SMC5-DDK-mini-AID (AID47) from the endogenous locus either as heterozygous or homozygous alleles. Moreover, 100 µM of Indole-3-acetic acid (IAA) was added to the culture media to deplete SMC5. RNAi silencing transfection to deplete CSN1 was performed as previously described [73].

### 4.2. DNA Fiber Assay

mESCs were treated 40–48 h after passage. mESCs were incubated in culture with 250 µM 5-chloro-2′-deoxyuridine (CldU, Sigma, C6891) for 20 min, washed twice with PBS, incubated with 2 mM hydroxyurea (HU, Sigma, H8627) for 3 h, washed twice with PBS, and incubated with 250 µM 5-Iodo-2′-deoxyuridine (IdU, Sigma I7125) for 20 min. Then, 100 µM IAA (Sigma, I5148), 50 µM mirin (Cayman, 13208), 10 µM PFM01 (Tocris, 622210, Bristol, UK), 10 µM DNA2-IN-C5 (Aobious, AOB9082, Gloucester, MA, USA), 2.5 µM CSN5i-3 (Novartis Pharma, Basel, Switzerland), 25 µM compound 33-11 (ChemBridge Corporation, 6655693, San Diego, CA, USA), or 5 µM ML216 (Aobious, AOB1300) were added at the indicated time points. In place of HU treatment, aphidicolin (Cayman Chemical, 14007) was used at 15 µM. Labeled mESCs were treated with TrypLE and resuspended in PBS at 2 × 10^5^ cells/mL. DNA fiber spreading and immunostaining were performed as previously described [137]. Primary antibodies used were rat anti-BrdU (CldU) (Abcam, Waltham, USA) and mouse anti-BrdU (IdU) (Becton Dickinson, Franklin Lakes, NJ, USA). Secondary antibodies used were Alexa Fluor anti-rat 568 and Alexa Fluor anti-mouse 488. Antibody information is presented in Supplementary Table S1.

### 4.3. mESC Immunocytochemistry

mESCs were treated with 20 µM 5-Ethynyl-2′-deoxyuridine (EdU, Sigma, T511285) and 2 mM HU, with or without 100 µM IAA or 2.5 µM CSN5i-3. After 3 h of treatment, the cells were collected and nuclear clearance was performed as previously described [79,80]. Briefly, mESCs in single-cell suspension were suspended in 1 mL of nuclear clearance buffer (1.1915 g HEPES, 2.92 g NaCl, 0.142 g MgCl, 0.19 g EGTA, 51.3 g Sucrose, 1 mL Triton, 1 g BSA, add diH2O for 500 mL) and incubated at room temperature for 8 min. mESCs were centrifuged at 200× *g*, followed by cell fixation and immunocytochemistry, as described previously [136]. For EdU detection, nuclear clearance preparations were washed three times in PBS and incubated with ‘click’ reaction cocktail containing 0.1 M Tris (pH 8.5), 10 µM cyanine 5-azide (Lumiprobe, 13030, Hunt Valley, MD, USA), 1 mM CuSO_4_, and 0.1 M L-ascorbic acid (Sigma, A7506) added last. All reaction components were dissolved in PBS. Slides were incubated with the reaction cocktail for 20 min and washed in PBS with 0.5% Triton three times for 10 min each. The antibodies used are listed in Supplementary Table S1. Samples were mounted using Vectashield with 4′,6-diamidino-2-phenylindole (DAPI, Vector Laboratories, H-1200, Newark, CA, USA).

### 4.4. Western Blot Analysis

Subcellular fractionation was performed as previously described [45]. Nuclear pellets were lysed in RIPA buffer (Santa Cruz Biotechnology, sc-24948A, Dallas, TX, USA). Equal amounts of protein were separated by SDS-PAGE and transferred to PVDF membranes (Bio-Rad, 1620177, Hercules, CA, USA). Primary and secondary antibody information is provided in Supplementary Table S1. We used horseradish peroxidase (HRP)-conjugated goat anti-mouse-IgG and anti-rabbit-IgG secondary antibodies (Invitrogen). The signal was detected using Clarity Western ECL Substrate (Bio-Rad, 170-5061) and imaged using the Syngene XR5 system and GeneSys V1.5.2 (Syngene, Bengaluru, Karnataka, India).

### 4.5. Microscopy

Images were captured using a Zeiss Cell Observer Z1 fluorescence microscope linked to an ORCA-Flash 4.0 CMOS camera (Hamamatsu, Shizuoka, Japan), or a Zeiss AxioImager A2 linked to an AxioCam ERc 5s camera (Zeiss, Oberkochen, Germany). Images were analyzed and processed using ZEN 2012 blue edition imaging software (V1.1.1.0, Zeiss). Photoshop V13.0 (Adobe, San Jose, CA, USA) was used to prepare figure images.

### 4.6. Image Data Quantification

For measurements of DNA fiber length, a line was drawn along each dual-labeled DNA fiber. The length of the line was measured. Image data quantification was performed using ImageJ V1.54f (National Institutes of Health, MD, USA) [138,139]. For the measurement of FANCD2, FANCM, and 53BP1 focal intensity in whole cells, the pixel intensity at a point in the center of an individual focus was calculated using ImageJ V1.54f. This was performed for three foci per cell, and the average of the three measurements was calculated to determine the average focal intensity in each cell. For the measurement of CSN1 intensity, the average pixel intensity within an outlined area corresponding to the nuclear boundary was calculated in each cell. All image data quantification was performed using ImageJ V1.54f.

### 4.7. Statistical Analysis

Statistical analyses were performed using RStudio 2021.09.4+403.pro3 and GraphPad Prism 9.5.1. A non-parametric unpaired two-tailed Mann–Whitney U-test or a chi-squared test with Yates’ continuity correction was used for all assessments. *p*-values of less than 0.05 were considered significant. All data represent the means ± S.E.M. unless noted otherwise. * = *p* < 0.05; ** = *p* < 0.01; *** = *p* < 0.001; **** = *p* < 0.0001; and ns (not significant) indicates >0.05. Individual *p*-values for all graphs presented in each figure are available in Supplementary Table S2.

## Data Availability

The data that support the findings of this study are available from the corresponding authors upon reasonable request.

## Supplementary Materials

The following supporting information can be downloaded at: https://www.mdpi.com/article/10.3390/ijms25020952/s1. Reference [140] are cited in the supplementary materials.

## Abbreviations

SMC	structural maintenance of chromosomes
NSMCE	non-SMC element
CSN	COP9 signalosome
CRL	cullin ring ligase
mESC	mouse embryonic stem cell
AID	auxin-inducible degron
ssDNA	single-stranded DNA
dsDNA	double-stranded DNA
HU	hydroxyurea
CldU	5-chloro-2′-deoxyuridine
IdU	5-iodo-2′-deoxyuridine
EdU	5-ethynyl-2′-deoxyuridine
IAA	indole-3-acetic acid
DAPI	4′,6-diamidino-2-phenylindole
RPA	replication protein A
RAD51	radiation sensitive 51
FA	Fanconi anemia
FANC	FA complementation group
ATR	ataxia telangiectasia and Rad3-related
SNF2	sucrose non-fermenting 2
SMARCAL1	SWI/SNF-related, matrix-associated, actin-dependent regulator of chromatin, subfamily A like 1
ZRANB3	zinc finger RANBP2-type containing 3
HLTF	Helicase-like transcription factor
FBH1	F-box DNA helicase 1
MRE11	meiotic recombination 11
EXO1	exonuclease 1
ABRO1	abraxas 2, BRISC complex subunit
BRCA	breast cancer
53BP1	p53 binding protein 1
VHL	Von Hippel–Lindau
BOD1L	bio-orientation defect 1-like
BLM	Bloom syndrome
NEDD8	neural precursor cell-expressed, developmentally down-regulated 8
HBx	hepatitis B virus protein X
SUMO	small ubiquitin-related modifier
S.E.M.	standard error of the mean
