# Supplementary material for: SMC5/6 Promotes Replication Fork Stability via Negative Regulation of the COP9 Signalosome
Source: Int J Mol Sci. 2024 Jan 12;25(2):952. doi: 10.3390/ijms25020952 (PMC10815603; doi:10.3390/ijms25020952)
Supplement: Supplementary file 1 [file ijms-25-00952-s001.zip › ijms-2782819-supplementary.pdf]

Supplementary Materials:

Supplementary Figure S1. SMC5/6 is required for replication fork restart following aphidicolin-mediated replication block.

Supplementary Figure S2. Fork restart defect upon SMC5/6 loss is MRE11-dependent during aphidicolin-mediated replication block and requires MRE11 endonuclease activity.

Supplementary Figure S3. SMC5/6 negatively modulates CSN protein levels.

Supplementary Table S1: Antibodies used in this study.

Supplementary Table S2: Statistical analyses performed in this study.

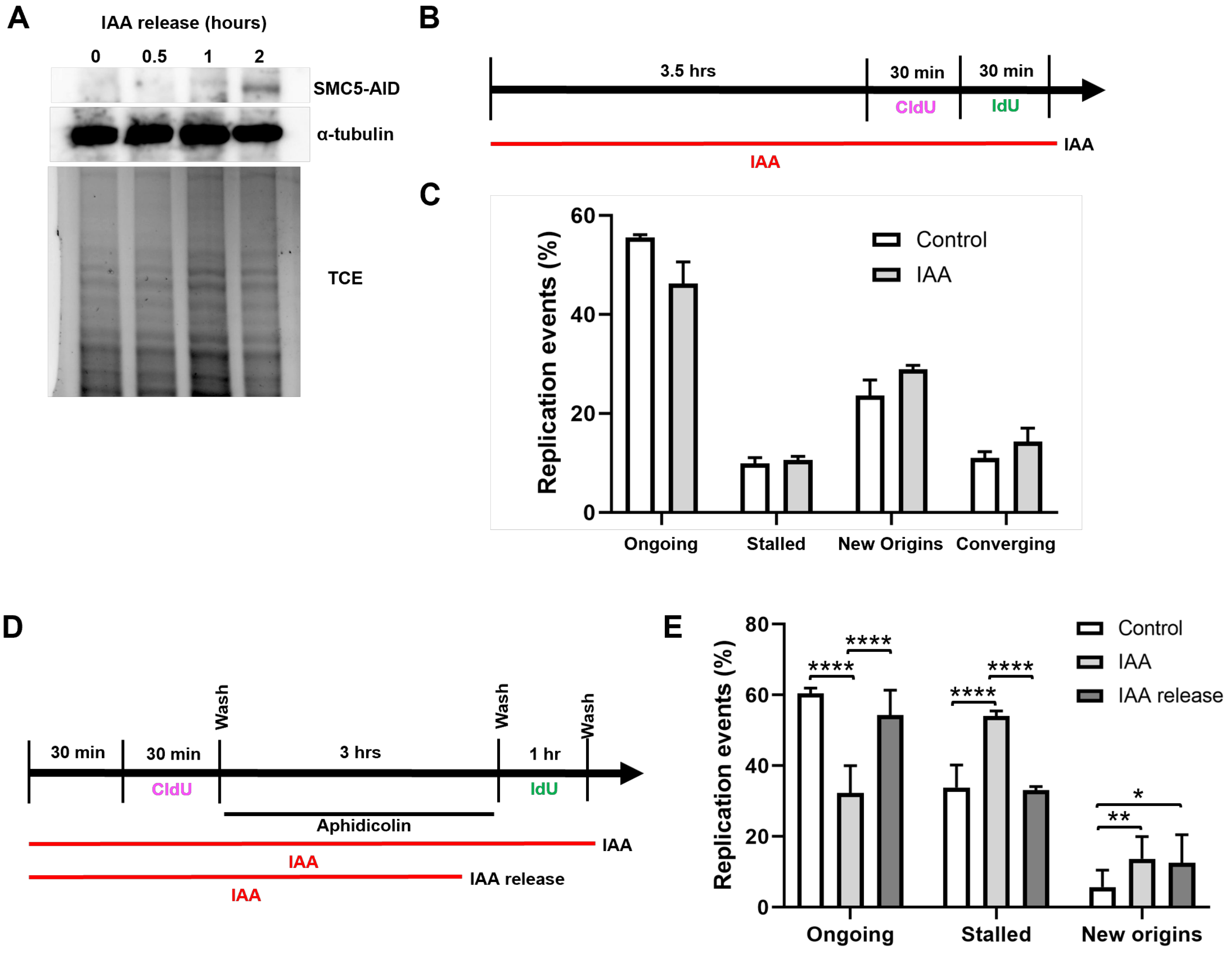

Supplementary Figure S1. SMC5/6 is required for replication fork restart following aphidicolin-mediated replication block. (**A**) Western blot analysis of SMC5-AID protein levels following IAA (100 μM) removal for 0, 0.5, 1, and 2 hours, in *Smc5-AID* homozygous mESCs. Alpha-tubulin was used as a loading control, and 2,2,2-Trichloroethanol (TCE) was incorporated in the gel to visualize total loaded protein (lower panel). (**B**) Schematic of CldU (250 μM) and IdU (250 μM) labeling following 3.5 hours of IAA treatment. (**C**) Quantification of replication event frequency in control and IAA conditions. Data represent mean ± S.E.M. (control condition: n = 235 fibers, IAA condition: n = 255 fibers). 3 experiments were performed for each condition. (**D**) Schematic of CldU (250 μM) and IdU (250 μM) labeling and aphidicolin (15 μM) and IAA treatment and release (**E**) Quantification of replication event frequency in control, IAA, IAA release, and IAA add conditions. Data represent mean ± S.E.M. (control condition: n = 276 fibers, IAA condition: n = 289 fibers, IAA release condition: n = 222 fibers). 3 experiments were

performed for each condition. Pearson's Chi-squared test with Yates' continuity correction, *$p<0.05$, **$p<0.005$, ****$p < 0.0001$. See Supplementary Table S2 for p-values and statistics.

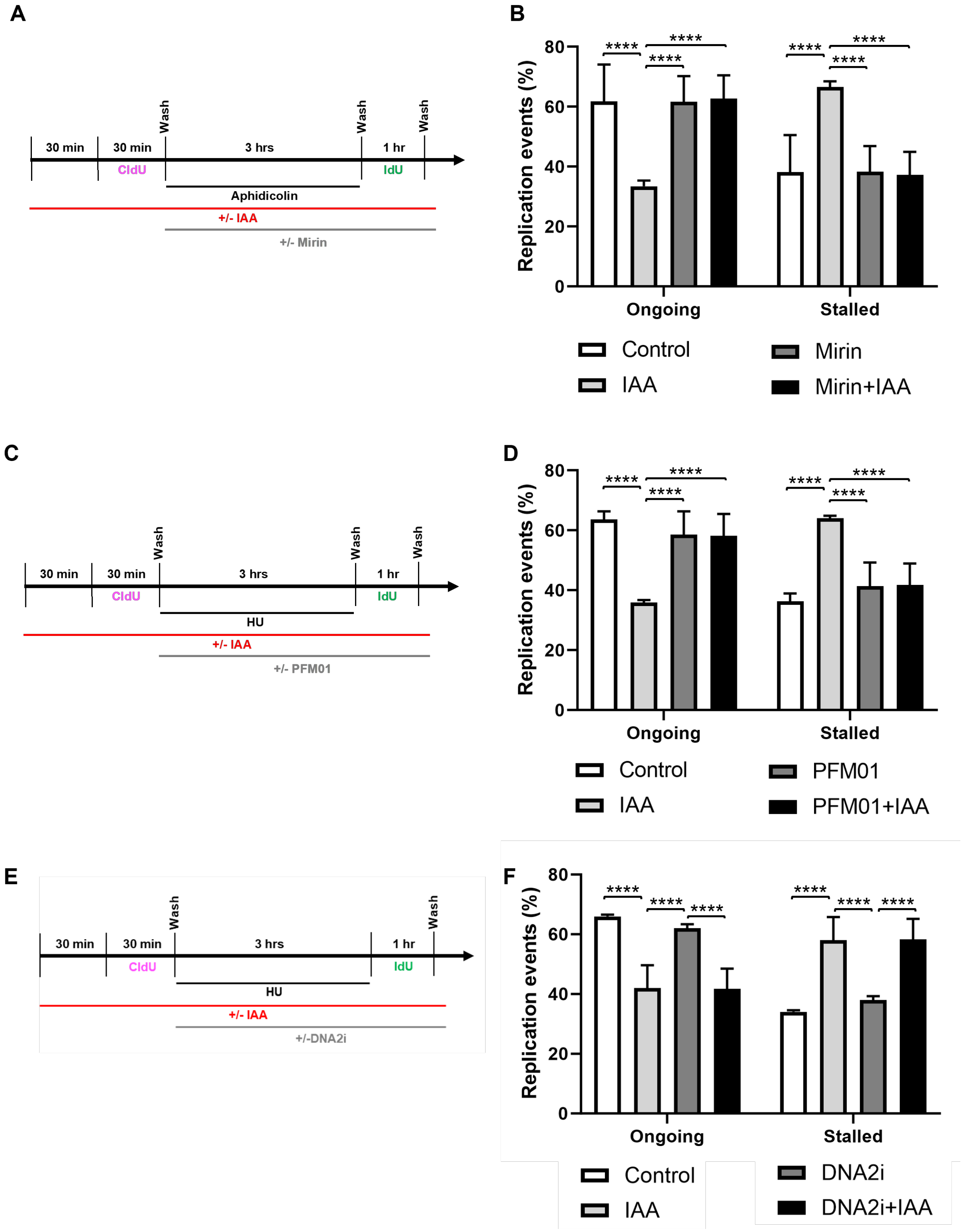

Supplementary Figure S2. Fork restart defect upon SMC5/6 loss is MRE11-dependent during aphidicolin-mediated replication block and requires MRE11 endonuclease activity. (**A**) Schematic of CldU and IdU labeling and aphidicolin, IAA, and mirin (50 μM) treatment. (**B**) Quantification of replication event frequency in control and IAA-treated mESCs, with or without mirin treatment. Data

represent mean ± S.E.M. (control condition: n = 498 fibers, mirin condition: n = 499 fibers, IAA condition: n = 500 fibers, mirin+IAA condition: n = 500 fibers). 3 experiments were performed for each condition. Pearson's Chi-squared test with Yates' continuity correction, ****p < 0.0001. (**C**) Schematic of CldU and IdU labeling and HU, IAA, and PFM01 (10 μM) treatment. (**D**) Quantification of replication event frequency in control and IAA-treated mESCs, with or without PFM01 treatment. Data represent mean ± S.E.M. (control condition: n = 409 fibers, PFM01 condition: n = 272 fibers, IAA condition: n = 294 fibers, PFM01+IAA condition: n = 338 fibers). 3 experiments were performed for each condition. Pearson's Chi-squared test with Yates' continuity correction, ****p < 0.0001. (**E**) Schematic of CldU and IdU labeling and HU, IAA, and DNA2i (10 μM) treatment. (**F**) Quantification of replication event frequency in control and IAA-treated mESCs, with or without DNA2i treatment. Data represent mean ± S.E.M. (control condition: n = 121 fibers, DNA2i condition: n = 170 fibers, IAA condition: n = 243 fibers, PFM01+IAA condition: n = 118 fibers). 3 experiments were performed for each condition. Pearson's Chi-squared test with Yates' continuity correction, ****p < 0.0001. See Supplementary Table S2 for p-values and statistics.

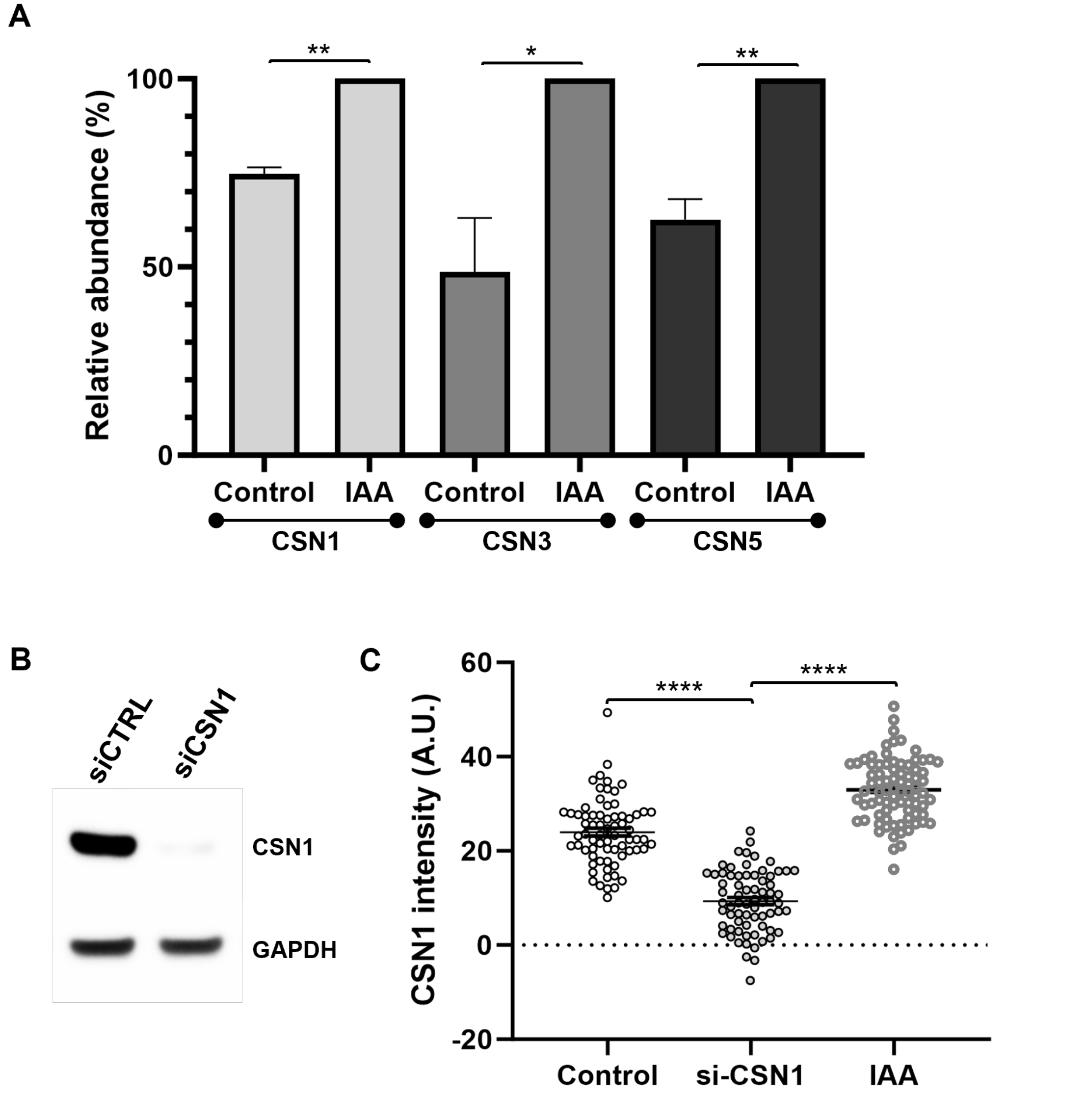

Supplementary Figure S3. SMC5/6 negatively modulates CSN protein levels. (**A**) Quantification of CSN1, CSN3, and CSN5 Western blots for control and IAA conditions using the Band/Peak Quantification Tool on Image J [140]. Western blots were performed in triplicate. Data represent mean ± S.E.M. A paired two-tailed t test, ** p < 0.005, * p < 0.05. (**B**) Western blot of assessment of CSN1 depletion following siRNA treatment. (**C**) Quantification of average CSN1 intensity in control, siCSN1, and IAA-treated *Smc5-AID* mESCs. Data represent mean ± S.E.M. (control condition: n = 70 cells, siCSN1 condition: n = 70 cells, IAA condition: n = 81 cells). Unpaired two-tailed Mann-Whitney test, ****p < 0.0001.

Supplemental Table S1: Antibodies used in this study

Primary Antibodies	Host	Source	Cat. Number	ICC Dilution	WB Dilution
53BP1	Rabbit	Abcam	ab21083	1 in 100	
alpha-tubulin	Mouse	ThermoFisher	T9026		1 in 1000
CldU	Rat	Abcam	ab6326	1 in 100	
CSN1	Rabbit	Fortis Life Sciences	A300-026A	1 in 100	1 in 1000
CSN3	Rabbit	Fortis Life Sciences	A300-012A		1 in 1000
CSN5	Rabbit	Fortis Life Sciences	A300-014A		1 in 1000
FANCD2	Rabbit	Novus	NB100-182	1 in 600	
FANCM	Rabbit	Abclonal	A7602	1 in 200	
H4K12ac	Mouse	Abcam	ab46983		
IdU	Mouse	Becton Dickinson	347580	1 in 40	
SMC5	Rabbit	Novus	100-469		1 in 400
**Secondary Antibodies**	**Host**	**Source**	**Cat. Number**	**ICC Dilution**	**WB Dilution**
Mouse IgG, Alexa Fluor 488	Goat	Invitrogen	A-11001	1 in 200	
Mouse IgG, Alexa Fluor 568	Goat	Invitrogen	A-11031	1 in 3000	
Rat IgG, Alexa Fluor 568	Goat	Invitrogen	A-11077	1 in 200	
Rabbit IgG, Alexa Fluor 488	Goat	Invitrogen	A-11008	1 in 3000	
Rabbit IgG, Alexa Fluor 568	Goat	Invitrogen	A-11011	1 in 3000	
Mouse IgG, HRP	Rabbit	Invitrogen	31450		1 in 10,000
Rabbit IgG, HRP	Goat	Invitrogen	31466		1 in 10,000

Supplemental Table S2: Statistics and p-value calculations.

P-values are defined as: * p < 0.05, ** p < 0.005, *** p < 0.0005, **** p < 0.0001

Figure Reference	Significance	p-value	Statistical test	Group
**Figure 2**				
**Figure 2D**				
Control versus IAA	****	<0.0001	Pearson's Chi-squared test with Yates' continuity correction	Ongoing
Control versus IAA release	n.s.	0.4129	Pearson's Chi-squared test with Yates' continuity correction	Ongoing
Control versus IAA add	*	0.012	Pearson's Chi-squared test with Yates' continuity correction	Ongoing
IAA versus IAA release	****	<0.0001	Pearson's Chi-squared test with Yates' continuity correction	Ongoing
IAA versus IAA add	****	<0.0001	Pearson's Chi-squared test with Yates' continuity correction	Ongoing
IAA release versus IAA add	**	0.0022	Pearson's Chi-squared test with Yates' continuity correction	Ongoing
Control versus IAA	****	<0.0001	Pearson's Chi-squared test with Yates' continuity correction	Stalled
Control versus IAA release	n.s.	0.3179	Pearson's Chi-squared test with Yates' continuity correction	Stalled
Control versus IAA add	***	0.0002	Pearson's Chi-squared test with Yates' continuity correction	Stalled
IAA versus IAA release	****	<0.0001	Pearson's Chi-squared test with Yates' continuity correction	Stalled
IAA versus IAA add	****	<0.0001	Pearson's Chi-squared test with Yates' continuity correction	Stalled
IAA release versus IAA add	**	0.0041	Pearson's Chi-squared test with Yates' continuity correction	Stalled
Control versus IAA	*	0.0315	Pearson's Chi-squared test with Yates' continuity correction	New origins
Control versus IAA release	n.s.	0.3634	Pearson's Chi-squared test with Yates' continuity correction	New origins
Control versus IAA add	n.s.	0.4991	Pearson's Chi-squared test with Yates' continuity correction	New origins
IAA versus IAA release	*	0.008	Pearson's Chi-squared test with Yates' continuity correction	New origins
IAA versus IAA add	*	0.0295	Pearson's Chi-squared test with Yates' continuity correction	New origins
IAA release versus IAA add	n.s.	0.226	Pearson's Chi-squared test with Yates' continuity correction	New origins
**Supplemental Figure S1**				
**Supplemental Figure S1B**				
Control versus IAA	****	<0.0001	Pearson's Chi-squared test with Yates' continuity correction	Ongoing
Control versus IAA release	n.s.	0.3772	Pearson's Chi-squared test with Yates' continuity correction	Ongoing
IAA versus IAA release	****	<0.0001	Pearson's Chi-squared test with Yates' continuity correction	Ongoing
Control versus IAA	****	<0.0001	Pearson's Chi-squared test with Yates' continuity correction	Stalled
Control versus IAA release	n.s.	0.121	Pearson's Chi-squared test with Yates' continuity correction	Stalled
IAA versus IAA release	****	<0.0001	Pearson's Chi-squared test with Yates' continuity correction	Stalled
Control versus IAA	**	0.0028	Pearson's Chi-squared test with Yates' continuity correction	New origins
Control versus IAA release	*	0.0164	Pearson's Chi-squared test with Yates' continuity correction	New origins
IAA versus IAA release	n.s.	0.5354	Pearson's Chi-squared test with Yates' continuity correction	New origins

Figure Reference	Significance	p-value	Statistical test	Group
**Figure 3**				
**Figure 3C**				
Control versus IAA	****	<0.0001	Unpaired two-tailed Mann-Whitney test	
**Figure 3E**				
Control versus IAA	****	<0.0001	Unpaired two-tailed Mann-Whitney test	
**Figure 3G**				
Control versus IAA	****	<0.0001	Unpaired two-tailed Mann-Whitney test	

Figure 4				
**Figure 4B**				
Control versus mirin	n.s.	0.4129	Pearson's Chi-squared test with Yates’ continuity correction	Ongoing
Control versus IAA	****	<0.0001	Pearson's Chi-squared test with Yates’ continuity correction	Ongoing
Control versus mirin+IAA	n.s.	0.3389	Pearson's Chi-squared test with Yates’ continuity correction	Ongoing
Mirin versus IAA	n.s.	0.1989	Pearson's Chi-squared test with Yates’ continuity correction	Ongoing
Mirin versus mirin+IAA	n.s.	0.6811	Pearson's Chi-squared test with Yates’ continuity correction	Ongoing
IAA versus mirin+IAA	****	<0.0001	Pearson's Chi-squared test with Yates’ continuity correction	Ongoing
Control versus mirin	n.s.	0.6005	Pearson's Chi-squared test with Yates’ continuity correction	Stalled
Control versus IAA	****	<0.0001	Pearson's Chi-squared test with Yates’ continuity correction	Stalled
Control versus mirin+IAA	n.s.	0.554	Pearson's Chi-squared test with Yates’ continuity correction	Stalled
Mirin versus IAA	n.s.	0.4414	Pearson's Chi-squared test with Yates’ continuity correction	Stalled
Mirin versus mirin+IAA	n.s.	0.2986	Pearson's Chi-squared test with Yates’ continuity correction	Stalled
IAA versus mirin+IAA	****	<0.0001	Pearson's Chi-squared test with Yates’ continuity correction	Stalled
**Figure 4D**				
Control versus IAA	***	0.0003	Unpaired two-tailed Mann-Whitney test	
Control versus mirin	n.s.	0.0639	Unpaired two-tailed Mann-Whitney test	
Control versus mirin+IAA	*	0.0441	Unpaired two-tailed Mann-Whitney test	
IAA versus mirin	****	<0.0001	Unpaired two-tailed Mann-Whitney test	
IAA versus mirin+IAA	****	<0.0001	Unpaired two-tailed Mann-Whitney test	
mirin versus mirin+IAA	n.s.	0.933	Unpaired two-tailed Mann-Whitney test	
**Figure 4F**				
Control versus IAA	****	<0.0001	Pearson's Chi-squared test with Yates’ continuity correction	Ongoing
Control versus ML216	*	0.0344	Pearson's Chi-squared test with Yates’ continuity correction	Ongoing
Control versus ML216+IAA	***	0.0001	Pearson's Chi-squared test with Yates’ continuity correction	Ongoing
ML216 versus IAA	***	0.0003	Pearson's Chi-squared test with Yates’ continuity correction	Ongoing
ML216 versus ML216+IAA	**	0.0036	Pearson's Chi-squared test with Yates’ continuity correction	Ongoing
IAA versus ML216+IAA	n.s.	0.651	Pearson's Chi-squared test with Yates’ continuity correction	Ongoing
Control versus ML216	*	0.0411	Pearson's Chi-squared test with Yates’ continuity correction	Stalled
Control versus IAA	****	<0.0001	Pearson's Chi-squared test with Yates’ continuity correction	Stalled
Control versus ML216+IAA	***	0.0002	Pearson's Chi-squared test with Yates’ continuity correction	Stalled
ML216 versus IAA	***	0.0001	Pearson's Chi-squared test with Yates’ continuity correction	Stalled
ML216 versus ML216+IAA	**	0.0012	Pearson's Chi-squared test with Yates’ continuity correction	Stalled
IAA versus ML216+IAA	n.s.	0.1929	Pearson's Chi-squared test with Yates’ continuity correction	Stalled

Supplemental Figure S2				
**Supplemental Figure S2B**				
Control versus IAA	****	<0.0001	Pearson's Chi-squared test with Yates' continuity correction	Ongoing
Control versus mirin	n.s.	0.4441	Pearson's Chi-squared test with Yates' continuity correction	Ongoing
Control versus mirin+IAA	n.s.	0.3998	Pearson's Chi-squared test with Yates' continuity correction	Ongoing
Mirin versus IAA	****	<0.0001	Pearson's Chi-squared test with Yates' continuity correction	Ongoing
Mirin versus mirin+IAA	n.s.	0.0808	Pearson's Chi-squared test with Yates' continuity correction	Ongoing
IAA versus mirin+IAA	****	<0.0001	Pearson's Chi-squared test with Yates' continuity correction	Ongoing
Control versus IAA	****	<0.0001	Pearson's Chi-squared test with Yates' continuity correction	Stalled
Control versus mirin	n.s.	0.4122	Pearson's Chi-squared test with Yates' continuity correction	Stalled
Control versus mirin+IAA	n.s.	0.322	Pearson's Chi-squared test with Yates' continuity correction	Stalled
Mirin versus IAA	****	<0.0001	Pearson's Chi-squared test with Yates' continuity correction	Stalled
Mirin versus mirin+IAA	n.s.	0.3998	Pearson's Chi-squared test with Yates' continuity correction	Stalled
IAA versus mirin+IAA	****	<0.0001	Pearson's Chi-squared test with Yates' continuity correction	Stalled
**Supplemental Figure S2D**				
Control versus IAA	****	<0.0001	Pearson's Chi-squared test with Yates' continuity correction	Ongoing
Control versus PFM01	n.s.	0.668	Pearson's Chi-squared test with Yates' continuity correction	Ongoing
Control versus PFM01+IAA	n.s.	0.3911	Pearson's Chi-squared test with Yates' continuity correction	Ongoing
PFM01 versus IAA	****	<0.0001	Pearson's Chi-squared test with Yates' continuity correction	Ongoing
PFM01 versus PFM01+IAA	n.s.	0.5112	Pearson's Chi-squared test with Yates' continuity correction	Ongoing
IAA versus PFM01+IAA	****	<0.0001	Pearson's Chi-squared test with Yates' continuity correction	Ongoing
Control versus IAA	****	<0.0001	Pearson's Chi-squared test with Yates' continuity correction	Stalled
Control versus PFM01	n.s.	0.3319	Pearson's Chi-squared test with Yates' continuity correction	Stalled
Control versus PFM01+IAA	n.s.	0.0919	Pearson's Chi-squared test with Yates' continuity correction	Stalled
PFM01 versus IAA	****	<0.0001	Pearson's Chi-squared test with Yates' continuity correction	Stalled
PFM01 versus PFM01+IAA	n.s.	0.3988	Pearson's Chi-squared test with Yates' continuity correction	Stalled
IAA versus PFM01+IAA	****	<0.0001	Pearson's Chi-squared test with Yates' continuity correction	Stalled

Figure Reference	Significance	p-value	Statistical test	Group
**Figure 5**				
**Figure 5C**				
Control versus IAA	****	<0.0001	Unpaired two-tailed Mann-Whitney test	
**Figure 5E**				
Control versus IAA	****	<0.0001	Pearson's Chi-squared test with Yates’ continuity correction	Ongoing
Control versus CSNi	****	<0.0001	Pearson's Chi-squared test with Yates’ continuity correction	Ongoing
Control versus CSNi+IAA	**	0.0025	Pearson's Chi-squared test with Yates’ continuity correction	Ongoing
Control versus CSNi+CUL4i	****	<0.0001	Pearson's Chi-squared test with Yates’ continuity correction	Ongoing
Control versus CSNi+CUL4i+IAA	****	<0.0001	Pearson's Chi-squared test with Yates’ continuity correction	Ongoing
IAA verses CSNi	n.s.	0.5449	Pearson's Chi-squared test with Yates’ continuity correction	Ongoing
IAA versus CSNi+IAA	***	0.0003	Pearson's Chi-squared test with Yates’ continuity correction	Ongoing
IAA versus CSNi+CUL4i	**	0.0044	Pearson's Chi-squared test with Yates’ continuity correction	Ongoing
IAA versus CSNi+CUL4i+IAA	n.s.	0.6566	Pearson's Chi-squared test with Yates’ continuity correction	Ongoing
CSNi versus CSNi+IAA	***	0.0003	Pearson's Chi-squared test with Yates’ continuity correction	Ongoing
CSNi versus CSNi+CUL4i	n.s.	0.3861	Pearson's Chi-squared test with Yates’ continuity correction	Ongoing
CSNi versus CSNi+CUL4i+IAA	*	0.0178	Pearson's Chi-squared test with Yates’ continuity correction	Ongoing
CSNi+IAA verses CSNi+CUL4i	***	0.0002	Pearson's Chi-squared test with Yates’ continuity correction	Ongoing
CSNi+IAA versus CSNi+CUL4i+IAA	**	0.0032	Pearson's Chi-squared test with Yates’ continuity correction	Ongoing
CSNi+CUL4i versus CSNi+CUL4i+IAA	**	0.0031	Pearson's Chi-squared test with Yates’ continuity correction	Ongoing
Control versus IAA	****	<0.0001	Pearson's Chi-squared test with Yates’ continuity correction	Stalled
Control versus CSNi	****	<0.0001	Pearson's Chi-squared test with Yates’ continuity correction	Stalled
Control versus CSNi+IAA	**	0.0022	Pearson's Chi-squared test with Yates’ continuity correction	Stalled
Control versus CSNi+CUL4i	****	<0.0001	Pearson's Chi-squared test with Yates’ continuity correction	Stalled
Control versus CSNi+CUL4i+IAA	***	0.0002	Pearson's Chi-squared test with Yates’ continuity correction	Stalled
IAA verses CSNi	*	0.0414	Pearson's Chi-squared test with Yates’ continuity correction	Stalled
IAA versus CSNi+IAA	**	0.0041	Pearson's Chi-squared test with Yates’ continuity correction	Stalled
IAA versus CSNi+CUL4i	****	<0.0001	Pearson's Chi-squared test with Yates’ continuity correction	Stalled
IAA versus CSNi+CUL4i+IAA	n.s.	0.4412	Pearson's Chi-squared test with Yates’ continuity correction	Stalled
CSNi versus CSNi+IAA	****	<0.0001	Pearson's Chi-squared test with Yates’ continuity correction	Stalled
CSNi versus CSNi+CUL4i	n.s.	0.6615	Pearson's Chi-squared test with Yates’ continuity correction	Stalled
CSNi versus CSNi+CUL4i+IAA	*	0.0241	Pearson's Chi-squared test with Yates’ continuity correction	Stalled
CSNi+IAA verses CSNi+CUL4i	***	0.0002	Pearson's Chi-squared test with Yates’ continuity correction	Stalled
CSNi+IAA versus CSNi+CUL4i+IAA	**	0.0032	Pearson's Chi-squared test with Yates’ continuity correction	Stalled
CSNi+CUL4i versus CSNi+CUL4i+IAA	**	0.0041	Pearson's Chi-squared test with Yates’ continuity correction	Stalled

Figure Reference	Significance	p-value	Statistical test	Group
**Figure 5G**				
Control versus IAA	****	<0.0001	Pearson's Chi-squared test with Yates’ continuity correction	Ongoing
Control versus CUL4i	****	<0.0001	Pearson's Chi-squared test with Yates’ continuity correction	Ongoing
Control versus CUL4i+IAA	****	<0.0001	Pearson's Chi-squared test with Yates’ continuity correction	Ongoing
Control versus mirin+CUL4i	*	0.0377	Pearson's Chi-squared test with Yates’ continuity correction	Ongoing
Control versus mirin+CUL4i+IAA	n.s.	0.1442	Pearson's Chi-squared test with Yates’ continuity correction	Ongoing
IAA versus CUL4i	n.s.	0.1333	Pearson's Chi-squared test with Yates’ continuity correction	Ongoing
IAA versus CUL4i+IAA	*	0.0098	Pearson's Chi-squared test with Yates’ continuity correction	Ongoing
IAA versus mirin+CUL4i	***	0.0002	Pearson's Chi-squared test with Yates’ continuity correction	Ongoing
IAA versus mirin+CUL4i+IAA	****	<0.0001	Pearson's Chi-squared test with Yates’ continuity correction	Ongoing
CUL4i versus CUL4i+IAA	n.s.	0.3122	Pearson's Chi-squared test with Yates’ continuity correction	Ongoing
CUL4i versus mirin+CUL4i	****	<0.0001	Pearson's Chi-squared test with Yates’ continuity correction	Ongoing
CUL4i versus mirin+CUL4i+IAA	****	<0.0001	Pearson's Chi-squared test with Yates’ continuity correction	Ongoing
CUL4i+IAA versus mirin+CUL4i	****	<0.0001	Pearson's Chi-squared test with Yates’ continuity correction	Ongoing
CUL4i+IAA versus mirin+CUL4i+IAA	****	<0.0001	Pearson's Chi-squared test with Yates’ continuity correction	Ongoing
mirin+CUL4i versus mirin+CUL4i+IAA	*	0.0473	Pearson's Chi-squared test with Yates’ continuity correction	Ongoing
Control versus IAA	****	<0.0001	Pearson's Chi-squared test with Yates’ continuity correction	Stalled
Control versus CUL4i	****	<0.0001	Pearson's Chi-squared test with Yates’ continuity correction	Stalled
Control versus CUL4i+IAA	****	<0.0001	Pearson's Chi-squared test with Yates’ continuity correction	Stalled
Control versus mirin+CUL4i	*	0.0169	Pearson's Chi-squared test with Yates’ continuity correction	Stalled
Control versus mirin+CUL4i+IAA	n.s.	0.3332	Pearson's Chi-squared test with Yates’ continuity correction	Stalled
IAA versus CUL4i	n.s.	0.3132	Pearson's Chi-squared test with Yates’ continuity correction	Stalled
IAA versus CUL4i+IAA	*	0.0311	Pearson's Chi-squared test with Yates’ continuity correction	Stalled
IAA versus mirin+CUL4i	***	0.0002	Pearson's Chi-squared test with Yates’ continuity correction	Stalled
IAA versus mirin+CUL4i+IAA	****	<0.0001	Pearson's Chi-squared test with Yates’ continuity correction	Stalled
CUL4i versus CUL4i+IAA	n.s.	0.1146	Pearson's Chi-squared test with Yates’ continuity correction	Stalled
CUL4i versus mirin+CUL4i	****	<0.0001	Pearson's Chi-squared test with Yates’ continuity correction	Stalled
CUL4i versus mirin+CUL4i+IAA	****	<0.0001	Pearson's Chi-squared test with Yates’ continuity correction	Stalled
CUL4i+IAA versus mirin+CUL4i	****	<0.0001	Pearson's Chi-squared test with Yates’ continuity correction	Stalled
CUL4i+IAA versus mirin+CUL4i+IAA	****	<0.0001	Pearson's Chi-squared test with Yates’ continuity correction	Stalled
mirin+CUL4i versus mirin+CUL4i+IAA	*	0.0415	Pearson's Chi-squared test with Yates’ continuity correction	Stalled

Figure Reference	Significance	p-value	Statistical test	Group
Figure 6				
**Figure 6C**				
Control versus IAA	****	<0.0001	Unpaired two-tailed Mann-Whitney test	
Control versus CSNi	n.s.	0.0787	Unpaired two-tailed Mann-Whitney test	
CSNi versus IAA	**	0.002	Unpaired two-tailed Mann-Whitney test	
**Figure 6E**				
Control versus IAA	****	<0.0001	Unpaired two-tailed Mann-Whitney test	
Control versus CSNi	n.s.	0.3718	Unpaired two-tailed Mann-Whitney test	
CSNi versus IAA	****	<0.0001	Unpaired two-tailed Mann-Whitney test	
